# Psychiatric Disorders after Epilepsy Diagnosis: A Population-Based Retrospective Cohort Study

**DOI:** 10.1371/journal.pone.0059999

**Published:** 2013-04-05

**Authors:** Hsiu-Ju Chang, Chien-Chang Liao, Chaur-Jong Hu, Winston W. Shen, Ta-Liang Chen

**Affiliations:** 1 School of Nursing, College of Nursing, Taipei Medical University, Taipei, Taiwan; 2 Health Policy Research Center, Taipei Medical University Hospital, Taipei, Taiwan; 3 Department of Anesthesiology, College of Medicine, Taipei Medical University, Taipei, Taiwan; 4 Department of Neurology, Taipei Medical University-Shuang Ho Hospital, New Taipei City, Taiwan; 5 College of Medicine, Taipei Medical University, Taipei, Taiwan; 6 Department of Psychiatry, Wan Fang Medical Center, affiliated with Department of Psychiatry, College of Medicine, Taipei Medical University, Taipei, Taiwan; University of South Florida, United States of America

## Abstract

**Background:**

Psychiatric manifestations after occurrence of epilepsy have often been noted. However, the association between newly diagnosed epilepsy and psychiatric disorders afterward is not completely understood. We conducted two longitudinal cohorts for patients with and without epilepsy to investigate the risk factors and hazard ratios of developing psychiatric disorders after patients were newly diagnosed with epilepsy.

**Methods:**

We identified 938 patients with a new diagnosis of epilepsy and 518,748 participants without epilepsy from the National Health Insurance Research Database in 2000–2002 and tracked them until 2008. We compared the incidence of developing psychiatric disorders between the two cohorts, evaluated risk factors and measured the associated hazard ratios (HRs) and 95% confidence intervals (CIs) of developing psychiatric disorders.

**Findings:**

The incidences of psychiatric disorders for people with and without epilepsy were 94.1 and 22.6 per 1000 person-years, respectively. After adjusting the covariates, the epilepsy cohort showed the highest risks in mental retardation (HR 31.5, 95% CI 18.9 to 52.4), bipolar disorder (HR 23.5, 95% CI 11.4 to 48.3) and alcohol or drug psychosis (HR 18.8, 95% CI 11.1 to 31.8) among psychiatric complications developed after newly diagnosed epilepsy. The risk increased with epileptic general seizure and frequency of outpatient visits for epilepsy, as well as with emergency room visits and hospitalizations for epilepsy, and with older age. Chronologically, the highest risk occurred in the first year after epilepsy diagnosis (HR 11.4, 95% CI 9.88 to 13.2).

**Conclusion:**

Various psychiatric disorders were demonstrated after newly diagnosed epilepsy and closely related to general seizure and use of medical services for epilepsy. This shows a need for integrated psychiatric care for patients newly diagnosed with epilepsy, especially in the first year.

## Introduction

Epilepsy is a common but serious neurological disease affecting an estimated 50 million people worldwide [1], with active epilepsy prevalence ranging from 0.2%–4.1% [2]. Epilepsy affects physical, mental and behavioral functions and is associated with higher risk of premature death due to traumatic brain injury [3], [4], status epilepticus, suicide, pneumonia and sudden death, and it accounts for 1.4% of all years of life lost [5], [6]. A study found 4.8% of patients with idiopathic generalized epilepsy (IGE) had attempted suicide, a much higher rate than that of the general population [7]–[9]. These suicidal patients were diagnosed with one or more psychiatric disorders [7]. Psychiatric disorders frequently affect 32% and 41% patients with epilepsy [10]–[12]. The most common psychiatric comorbidities found among patients with epilepsy are depression, neuroses (nonpsychotic anxiety disorders) and psychoses. These lead to a poorer prognosis for epileptic patients than for epileptic patients without psychiatric comorbidities [12], [13]. Special attention should be placed on depression because of its association with suicide risk in people with epilepsy [14].

Chronologically, psychiatric disorders may occur prior to, around the time of or following diagnosis of epilepsy [15]. Coexisting psychiatric conditions might complicate epilepsy patients’ diagnosis and treatment, worsen prognosis, increase health care service use and pose substantial worldwide socioeconomic burdens due to long-term disability, dependency and mortality [16]. This close relationship between epilepsy and psychiatric disorders highlights the importance of exploring risk factors of psychiatric disorders and their temporal relationships in patients with epilepsy.

Previous research was dominated by cross-sectional studies exploring the prevalence of various psychiatric disorders among patients with epilepsy. Neither retrospective studies showed the global features of psychiatric disorders that developed soon after epilepsy diagnosis, nor did existing studies specify the time sequence (onset) when psychiatric disorders developed in patients recently diagnosed with epilepsy. This study examines the incidence, hazard ratios and risk factors of a full spectrum of psychiatric disorders in patients newly diagnosed with epilepsy.

## Methods

### Study Design and Sample

This study used reimbursement claims from the Taiwan National Health Insurance program, which was implemented in March 1995 by incorporating the pre-existing 13 insurance programs and has come to provide coverage for more than 99% of Taiwan’s 23 million residents. Insurance claim data were obtained from the National Health Research Institutes with authorization from the Bureau of National Health Insurance of Taiwan’s Department of Health. The information available for this study included gender, birthdates, disease codes, health care rendered, medications prescribed, admissions, discharges, medical institutions and physicians providing services. For data analysis, we retrieved information about patient characteristics and medical care records by linking ambulatory claims, inpatient care claims and the registry for beneficiaries. Personal identification numbers were scrambled to protect patient privacy. The National Health Insurance Research Database was evaluated by a previous study [17] and was also used in our previous studies [18], [19].

We identified all patients aged ≥20 years with a new diagnosis of epilepsy in 2000–2002 as the case cohort. The reference cohort included people aged ≥20 years without diagnosis of epilepsy during the same period. Excluded from the study population were patients with any previous diagnosis of psychiatric disorder; this exclusion seeks to assure that participants were free from psychiatric disorders at the start of both cohorts. Overall, 519,686 insured adult beneficiaries were included in the longitudinal analysis. This follow-up started January 1, 2000, until censoring due to death or loss to follow-up by December 31, 2008, to explore whether individuals with new-onset epilepsy were associated with increased risk of developing psychiatric disorders.

### Criteria and Definition

We used *the International Classification of Diseases, 9th Revision, Clinical Modification* (ICD-9-CM) to identify individual health status including epilepsy (ICD-9-CM 345), psychiatric disorders (ICD-9-CM 290–319), asthma (ICD-9-CM 493), migraine (ICD-9-CM 346), stroke (ICD-9-CM 430–438), diabetes (ICD-9-CM 250), traumatic brain injury (ICD-9-CM 800–804, 850–854), brain tumor (ICD-9-CM 191, 225.0, 225.1, 225.2), cerebral palsy (ICD-9-CM 343), Parkinson’s disease (ICD-9-CM 332), Alzheimer’s disease (ICD-9-CM 331.0), congenital cardiac abnormalities (ICD-9-CM 745–746), pneumonia (ICD-9-CM 480–486) and gastrointestinal bleeding (ICD-9-CM 578.0, 578.1) [12], [18]–[21].

Epilepsy was categorized as general seizure including patients diagnosed as ICD-9 345.1, 345.3, 345.4, 345.7 and 345.61; the rest of ICD-9-CM 345 diagnoses were coded as partial seizure. To validate the potential impact of different medical service use among epileptic patients upon developing psychiatric disorders, we categorized the frequency of epilepsy-related, unrestricted and non-referral outpatient visits into quartiles as 1, 2–3, 4–16, and 17 medical visits within the follow-up period with primary diagnosis of epilepsy. We further evaluated patients with history of admission for emergency services or hospitalization with primary diagnosis as epilepsy within the follow-up period. Low income was defined as patients with certification of waived medical copayment. Population density or urbanization (persons/km^2^) was determined by dividing the nation’s 359 township and city district populations by the area (km^2^) of each administrative unit; these units were categorized from low to high into quartiles representing low, moderate, high and very high urbanization [22].

### Data Analysis

We used chi-square tests to compare the distribution of sociodemographic factors and pre-existing disease between the cohorts with and without epilepsy. Adjusted hazard ratios (HRs) with 95% confidence intervals (CIs) for epilepsy associated with psychiatric disorder risk were calculated using multivariate Cox proportional hazard analyses. All hazard ratios were initially adjusted for demographic variables including sex, age, income and urbanization. Potential medical variables that have been associated with epilepsy and psychiatric disorders such as asthma, migraine, stroke, diabetes, traumatic brain injury, brain tumor, cerebral palsy, Parkinson’s disease, Alzheimer’s disease, congenital cardiac abnormalities, pneumonia and gastrointestinal bleeding were added to adjust the hazard ratios [12]. Incidence of psychiatric disorders and specific psychiatric illness after epilepsy were also calculated [23]. We analyzed the data with Statistical Analysis System software version 9.1 (SAS Institute Inc., Carey, North Carolina, USA). A two-sided probability value of <0.05 was considered significant for the differences between groups.

### Ethical Approval

Insurance reimbursement claims used in this study were from Taiwan’s National Health Insurance Research Database, which is available for public access. This study was conducted in accordance with the Helsinki Declaration. To protect personal privacy, the electronic database was decoded with patient identifications scrambled for further public access for research. According to National Health Research Institute regulations, informed consent is not required due to decoded and scrambled patient identification. However, this study was approved by Taiwan’s National Health Research Institutes.

## Results

### Patient Demographic Characteristics

Characteristics of study groups with and without epilepsy were shown in **Table 1**. This study consisted of 938 persons with epilepsy and 518,748 persons without epilepsy after excluding ineligible subjects. The epilepsy cohort was older than the non-epilepsy reference group (45.3±17.6 years vs. 39.7±14.6 years, p<0.0001). The incidence of males (62.2% vs. 52.2%, p<0.0001) and patients with low income (6.0% vs. 1.6%, p<0.0001) was higher in the epilepsy cohort, and the epilepsy cohort had a higher rate of asthma (8.4% vs. 5.7%, p<0.001), diabetes (14.7% vs. 8.3%, p<0.0001), migraine (5.4% vs. 3.0%, p<0.0001), stroke (16.4% vs. 1.9%, p<0.0001), traumatic brain injury (15.6% vs. 4.1%, p<0.0001), brain tumor (3.7% vs. 0.2%, p<0.0001), cerebral palsy (1.2% vs. 0.3%, p<0.0001), Parkinson’s disease (2.4% vs. 0.3%, p<0.0001), Alzheimer’s disease (0.2% vs. 0.03%, p<0.01) and pneumonia (9.8% vs. 4.9%, p<0.001).

**Table 1:** Basic characteristics of study population of reference and epilepsy cohort.

	Reference		Epilepsy		
	N = 518748		N = 938		
	n	(%)	n	(%)	*p*-value
Male	270622	(52.2)	583	(62.2)	<0.0001
Age, years					<0.0001
20–29	152782	(29.5)	235	(25.1)	
30–39	137549	(26.5)	174	(18.6)	
40–49	112056	(21.6)	174	(18.6)	
50–59	56714	(10.9)	136	(14.5)	
60–69	34916	(6.7)	113	(12.1)	
≥70	24731	(4.8)	106	(11.3)	
Mean±SD	39.7±14.6		45.3±17.6		<0.0001
Low income	8202	(1.6)	56	(6.0)	<0.0001
Low urbanization	131133	(25.3)	258	(27.5)	0.24
Coexisting medical conditions					
Asthma	29748	(5.7)	79	(8.4)	0.0004
Diabetes	42899	(8.3)	138	(14.7)	<0.0001
Migraine	15497	(3.0)	51	(5.4)	<0.0001
Stroke	9910	(1.9)	154	(16.4)	<0.0001
Traumatic brain injury	21208	(4.1)	146	(15.6)	<0.0001
Brain tumor	1092	(0.2)	35	(3.7)	<0.0001
Cerebral palsy	1306	(0.3)	11	(1.2)	<0.0001
Parkinson’s disease	1513	(0.3)	22	(2.4)	<0.0001
Alzheimer’s disease	152	(0.03)	2	(0.2)	0.0011
Congenital cardiac abnormalities	707	(0.1)	2	(0.2)	0.52
Pneumonia	25484	(4.9)	92	(9.8)	<0.0001
Gastrointestinal bleeding	2065	(0.4)	4	(0.4)	0.89

### Incidence and Hazard Ratio of Psychiatric Disorders

**Table 2** lists adjusted HRs and 95% CIs for psychiatric disorders associated with epilepsy. The follow-up results show the psychiatric disorders with the highest incidence in patients newly diagnosed with epilepsy were neurotic disorders (44.7 per 1000 person-years), dementia, depression, alcohol and drug dependence, mental retardation, non-organic sleep disorders, alcohol and drug psychosis, schizophrenia, bipolar disorders, physiological malfunction, personality disorder and adjustment reaction. Epilepsy patients were 4.05 times more likely than non-epilepsy patients to develop psychiatric disorders (94.1 versus 22.6 per 1000 person-years), with a HR of 4.05 (95% CI 3.69 to 4.44) after adjustment for sociodemographic factors and pre-existing comorbidities. This was similar to the HR of 3.97 (95% CI 3.62 to 4.36) when adjusting for sociodemographic factors only. After adjusting for both sociodemographic and comorbidities covariates, the epilepsy cohort showed the highest risk for mental retardation (adult type, aged ≥20 and newly diagnosed after epilepsy) with a HR of 31.5 (95% CI 18.9 to 52.4) compared with the non-epilepsy reference cohort, followed by bipolar disorder (HR 23.5, 95% CI 11.4 to 48.3), alcohol or drug psychosis (HR 18.8, 95% CI 11.1 to 31.8) and schizophrenia (HR 12.1, 95% CI 6.79 to 21.6).

**Table 2:** Adjusted hazard ratios and 95% confidence intervals for risks of developing psychiatric disorders after epilepsy diagnosis.

		Reference cohort (N = 518748)		Epilepsy cohort (N = 938)		Adjusted risk	
	ICD-9-CM	Cases	Incidence^a^	Cases	Incidence^a^	HR	(95% CI)^b^
Psychiatric disorders	290–319	81114	22.6	446	94.1	4.05	(3.69–4.44)
Mental retardation^c^	317–319	341	0.10	18	3.80	31.5	(18.9–52.4)
Bipolar	296.4–296.7	269	0.07	8	1.69	23.5	(11.4–48.3)
Alcohol or drug psychosis	291, 292	521	0.15	15	3.16	18.8	(11.1–31.8)
Schizophrenia	295	729	0.20	12	2.53	12.1	(6.79–21.6)
Depression	296.2, 296.3	2754	0.77	27	5.69	7.16	(4.87–10.5)
Personality disorder	301	138	0.04	1	0.21	6.33	(0.88–45.6)
Dementia	290	2545	0.71	49	10.3	6.11	(4.59–8.14)
Neurotic disorders	300	33355	9.30	212	44.7	5.24	(4.57–6.00)
Non-organic sleep disorders	307.4	5896	1.64	17	3.59	2.37	(1.47–3.82)
Alcohol and drug dependence	303–305	9549	2.66	19	4.01	1.51	(0.96–2.37)
Physiological malfunction	306	5974	1.67	6	1.27	0.85	(0.38–1.88)
Adjustment reaction	309	1859	0.52	1	0.21	0.47	(0.07–3.35)
Other psychiatric illness^d^		17184	4.79	61	12.9	2.42	(1.88–3.11)

a Incidence presented as per 1000 person-years.

b Adjusted for age, sex, low income, urbanization, asthma, diabetes, migraine, stroke, traumatic brain injury, brain tumor, cerebral palsy, Parkinson’s disease, Alzheimer’s disease, congenital cardiac abnormalities, pneumonia and gastrointestinal bleeding.

c Mental retardation: adult type, aged ≥20 and newly diagnosed after epilepsy.

d Included ICD-9-CM 290–319 except for 290–292, 295, 296.4–296.7, 300, 301, 303–305, 306, 307.4, 309 and 317–319.

ICD-9-CM, International Classification of Diseases, 9th Revision, Clinical Modification.

### Role of Medical Care for Epilepsy

**Table 3** shows the relative risks and 95% CIs for psychiatric disorders in study participants according to seizure condition and history of epilepsy-related medical service use. Compared with patients without epilepsy, the relative risk of developing psychiatric disorders was 3.81 times higher (95% CI 3.40 to 4.27) in epileptic patients with partial seizure and 4.64 times higher (95% CI 3.94 to 5.45) in epileptic patients with general seizure. The risk of psychiatric disorders was associated with increased frequency of epilepsy-related outpatient visits during the follow-up period, with HRs of 3.74 (95% CI 3.17 to 4.41), 3.84 (95% CI 3.16 to 4.68), 4.00 (95% CI 3.22 to 4.98), and 4.72 (95% CI 3.96 to 5.64) for one, 2–3, 4–16, and ≥17 medical visits, respectively, compared to patients without epilepsy. The risk of psychiatric disorders tended to be higher for the group with a history of emergency care for epilepsy (HR 4.33, 95% CI 3.55 to 5.29) compared with epilepsy patients without emergency visits for epilepsy care (HR 3.98, 95% CI 3.58 to 4.42). The HRs of psychiatric disorders for epilepsy patients without and with history of hospitalization due to epilepsy were 3.78 (95% CI 3.40 to 4.19) and 5.56 (95% CI 4.54 to 6.81), respectively, compared with non-epilepsy patients.

**Table 3:** Risk of developing psychiatric disorders according to history of medical care for epilepsy.

	Number	HR	(95% CI)^a^
Seizure condition			
Without epilepsy	518748	1.00	(reference)
Epilepsy with partial seizure	650	3.81	(3.40–4.27)
Epilepsy with general seizure	288	4.64	(3.94–5.45)
Number of outpatient visits for epilepsy			
0 (without epilepsy)	518748	1.00	(reference)
1	311	3.74	(3.17–4.41)
2–3	218	3.84	(3.16–4.68)
4–16	175	4.00	(3.22–4.98)
≥17	234	4.72	(3.96–5.64)
Emergency visit for epilepsy			
Without epilepsy	518748	1.00	(reference)
Epilepsy without emergency care	742	3.98	(3.58–4.42)
Epilepsy with emergency care	196	4.33	(3.55–5.29)
Hospitalization for epilepsy			
Without epilepsy	518748	1.00	(reference)
Epilepsy without hospitalization	780	3.78	(3.40–4.19)
Epilepsy with hospitalization	158	5.56	(4.54–6.81)

a Adjusted for age, sex, low income, urban residence, asthma, diabetes, migraine, stroke, traumatic brain injury, brain tumor, cerebral palsy, Parkinson’s disease, Alzheimer’s disease, congenital cardiac abnormalities, pneumonia and gastrointestinal bleeding.

CI, confidence interval; HR, hazard ratio.

### Chronology of Developing psychiatric Disorders

**Figure 1:**
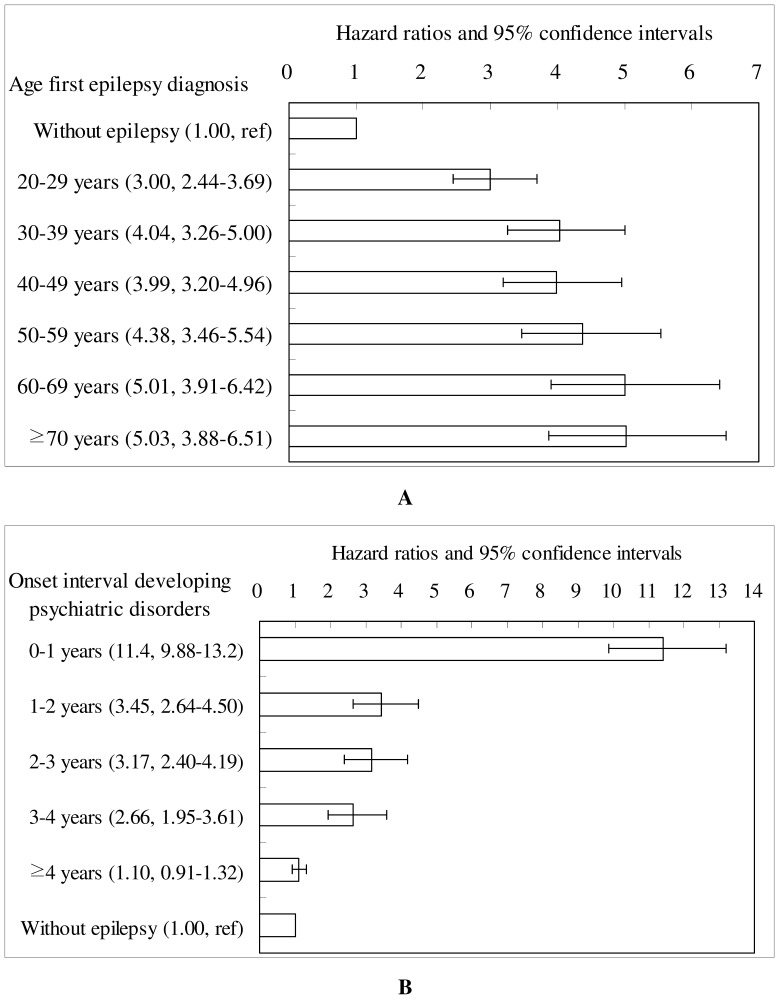
Hazard ratios and 95% confidence intervals of (A) age epilepsy diagnosed and (B) onset interval developing psychiatric disorders afterward (adjusted for age, sex, low income, urbanization, and coexisting medical conditions).

**Figures 1A and 1B** represented HRs and 95% CIs for developing psychiatric disorders in relation to age at first medical care and time since first diagnosis for epilepsy. Epilepsy was associated with increased risk of developing psychiatric disorders when patient age increases, and was highest in patients aged 70 and above (HR 5.03, 95% CI 3.88 to 6.51). The risk of developing psychiatric disorders was highest in the first year after epilepsy diagnosis (HR 11.4, 95% CI 9.88 to 13.2); this risk gradually diminished to non-significance after four years.

## Discussion

After exclusion of pre-existing psychiatric disorders and adjusting the associated covariates, this longitudinal cohort for epilepsy illustrated the significantly higher hazard ratio for mental retardation, bipolar disorders, alcohol and drug psychosis, schizophrenia, depression, personality disorders, dementia, neurotic disorders and non-organic sleep disorders among patients with newly diagnosed epilepsy when compared with control group. Risk determinants, including general seizure, frequency of epilepsy-related outpatient visits, history of using emergency services or hospitalization for epilepsy, age at epilepsy diagnosis and chronological incidence of developing psychiatric disorders after epilepsy diagnosis all contributed significantly to the incidence of developing psychiatric disorders after epilepsy.

The most prevalent psychiatric disorders have previously been found to be co-existing with (including prior to, at the time of, or following) epilepsy were depression, nonpsychotic anxiety disorders and psychoses, which was partially consistent with our findings [14], [15]. Prevalence estimates for mood and psychotic disorders due to general medical conditions are common. It has been observed that 25%–40% of individuals with certain neurological conditions such as epilepsy will develop a marked depressive disturbance and psychotic symptoms at some point during the course of illness [9], [10], [14], [15], [24]. After meticulous adjustments with exclusion of pre-existing psychiatric disorders before patients were diagnosed with epilepsy, we found the highest hazard ratio for mental retardation, followed by bipolar disorders, alcohol and drug psychosis, schizophrenia, depression, personality disorder, dementia, neurotic disorders and non-organic sleep disorders among epileptic patients when compared with control group. After an average latency of 10.5 years, 26.4% of children with epilepsy have been found to have varied levels of mental retardation [8]. Mean age of the adult group in our study developing mental retardation after epilepsy is 37.8 years, and our study clearly validated epileptic adults’ risk of developing mental retardation.

Mood disorders and depression were most frequently encountered and studied in patients with epilepsy [9]. Despite previous studies that found depression was one of the most common psychiatric disorders in patients with epilepsy, our study revealed that the HR of developing depression was not as high as that of mental retardation and bipolar disorders. Due to the influence of traditional illness beliefs in different cultures, patients may present depression in the form of somatic symptoms [25]. The influence of culture on the expression of depressive symptoms complicates the diagnosis of depressive disorders in such cases. Non-Western cultures such as Taiwan tend to emphasize the somatic symptoms of depression because the stigma attached to mental disorders leads patients to fear expressing their emotions [26], [27]. Compared with other countries, national community surveys in Taiwan have found relatively low depression rates [2]. The DSM-IV includes a range of somatic symptoms for the diagnosis of depression [24]. Although these symptoms are almost always an important part of depressive presentations, they may overlap with physical symptoms of epilepsy, and this could lead to underestimates of true depressive disorders in patients with epilepsy. Another influence is Chinese culture’s great emphasis on filial piety, which researchers have linked to less frequent depressive symptoms [28]. Chinese parents tend to behave toward their children in ways that are over-controlling, authoritarian and harsh. Strong filial piety beliefs advocating discipline and order in the family system as well as respect for and tolerance of parents may serve as a buffer against potential psychological harm [28]. To more accurately diagnose depression, strategies including the use of experienced assessors who employ a culturally sensitive approach in clinical practice may increase help-seeking behavior [25].

There is limited information about the incidence of bipolar disorders in epilepsy [29]. Study results varied depending on methodology used for screening as well as the selection criteria for the study sample for bipolar disorder. Although bipolar disorder had been reported to be rare among patients with epilepsy [30], it was found in 12.2% of epilepsy patients in a recent study [23]. The relative risk of bipolar symptoms among epilepsy patients is 1.6 to 2.2 times higher than among patients with other chronic conditions, and is 6.6 times higher than in healthy controls [23]. Mula et al. examined a sample of 143 adult outpatients with epilepsy and found that 11.8% met a DSM-based diagnosis of bipolar disorder, with only 1.4% considered to have “pure” bipolar disorder [29]. Clarke et al. showed the risk of bipolar disorders among epileptic individuals is 6.3 times higher than among individuals without epilepsy [31]. In line with recent studies, our results showed a HR of 23.5 for bipolar disorder, which was ranked after mental retardation in epilepsy patients compared with control group, suggesting a more robust relationship with epilepsy than previously considered. There are several possible explanations for this. First, a strong relationship between parent history of epilepsy and the development of psychosis for their offspring was found, although our data could not verify this [31]. Some cases might reflect such parent-child relationships, so the HR for bipolar disorder could have been higher than in other studies. Second, bipolar symptoms in patients with epilepsy often related to phenotype copies of bipolar disorder (such as interictal dysphoric disorder), preictal dysphoria, postictal mania, and anti-epileptic drugs [29]. Epilepsy, anxiety, depression and bipolar disorders often share common symptoms [32]. Our finding highlights the importance of screening for bipolar symptoms in patients with epilepsy to ensure proper psychiatric treatment.

Another interesting but controversial finding is that the relative risk of developing psychiatric disorders in epileptic patients with general seizure was higher than in epileptic patients with partial seizure compared with patients without epilepsy. Previous studies have found that partial seizures are a risk factor for developing anxiety and depression [33], [34]. The possible reason may be that partial seizures are often caused by organic lesions such as head injury, stroke, tumor and brain infection [35]. These risk factors related to partial seizure were controlled by our study so that the relative risk in developing psychiatric disorders was slightly lower than in general seizure cases. Epilepsy also has been highly correlated with substance abuse [36], schizophrenia or schizophrenia-like psychosis [37], personality disorders [38], neurotic disorders [12] and non-organic sleep disorders [11]. All those findings were also confirmed in our data.

To avoid bias, we used a large-scale retrospective longitudinal cohort study to explore the comprehensive features of psychiatric disorders after epilepsy diagnosis. The differences in study design and findings between Gaitatzis’ and ours are significant [15]. In our study, we focused on psychiatric disorders that emerged only after patients were newly diagnosed with epilepsy, and excluded patients with previous diagnosis of psychiatric disorders to ensure that participants were free from psychiatric disorders at the start of cohort. We suggest that this retrospective longitudinal observation of the risk of developing psychiatric disorders further strengthens the association between epilepsy and psychiatric disorders and enhances understanding of the role of epilepsy in the occurrence of psychiatric disorders.

Our study showed a high correlation between psychiatric disorders and epilepsy. We speculate that there are at least three plausible mechanisms for this correlation. First, psychiatric disorders might share common neurological pathogenic pathways with epilepsy, facilitating the occurrence of one in the presence of the other [39]. Depression has been related to neurological conditions such as dementia, which also accounts for epilepsy and is commonly seen in patients with learning difficulties [14]. Second, epilepsy and psychiatric disorders might relate to psychosocial stress [15]. Research has showed that depression is increased significantly after epilepsy and that depressed people have a higher risk of developing epilepsy [40], [41]. The bi-directional relationship between depression and epilepsy also supports the likelihood of potential mechanisms overlapping between them [42], [43]. Use of older antidepressant drugs has been associated with negative complications such as stroke [44], [45], which further complicates diagnosis and treatment. A previous study found the prevalence of anxiety in people with epilepsy relates to a current history of depression [46], further showing the close relationship between anxiety and depression in patients with epilepsy. The third possible mechanism is that every epileptic convulsion could induce brain ischemia and inflammation, resulting in subtle brain damage that might accumulate and cause psychiatric disorders. A high correlation has been found between seizures and substance abuse [36], and recent heroin use and alcohol consumption may be a further risk factor for seizure development [47], [48]. The psychosocial stress from epilepsy may result in substance abuse, constructing a more complicated pathogenic network.

Previous research showed that most epileptic patients use more health care services than people without epilepsy for a wide range of diseases, with the greatest proportion of patients with epilepsy consulting for psychiatric disorders [49]. Regarding medical care use as a predictor, a previous study showed that patients who visited neurology clinics have much higher rates of both anxiety and depression than those without clinic visits [50]. Frequent hospitalizations due to epilepsy also contribute significantly to depression [33]. This finding is potentially associated with observations that a more severe course of epilepsy contributes to emergence of depressive symptoms [51]. Our study demonstrated quantitatively with different hazard ratios that epileptic patients who visited outpatient clinics, used emergency services and were hospitalized more frequently had higher risks of developing psychiatric disorders. We also found that greater age at diagnosis of epilepsy related to higher risk of psychiatric disorders. Another study has also shown that the relative risk of depression among individuals with epilepsy is higher in older individuals compared to those without epilepsy [52].

Our study’s unique advantage is inclusion of a large population-based epilepsy cohort with case-control design. While most studies used instruments or telephone surveys, our study used reimbursement claims with ICD-9-CM codes as criteria for case selection. We also excluded any diagnosis of psychiatric disorders before the inclusion period to establish a more accurate relationship between psychiatric disorders and epilepsy. This retrospective cohort study analyzing the risk of developing psychiatric disorders after epilepsy enables greater understanding in clinical scenarios and suggests integrated care for epileptic patients. In addition, results related to a full spectrum of psychiatric disorders in newly diagnosed epilepsy patients confirmed a number of findings of previous independent investigations.

### Implications of this Study

Our study found that the first year after initial diagnosis of epilepsy had the highest hazard ratio of developing psychiatric disorders, with about 42.6% of new-onset psychiatric disorder cases clustering in this period, followed by the next highest incidence in the second year. Accurate assessment of and early intervention to treat psychiatric disorders, such as psychiatric consultation or integrative care by psychiatric specialists with neurologists, should be recommended routinely and delivered by health professionals during the first year after a diagnosis of epilepsy. Our study pointed out the contributory factors from clinical aspects, affecting the incidence of psychiatric disorders after new epilepsy diagnoses such as general seizure, frequency of epilepsy-related outpatient visits, emergency and in-hospital care for epilepsy, older age at diagnosis for epilepsy, and the first year after epilepsy diagnosis. These factors should be considered by health care providers to improve assessment, diagnosis and early treatment.

### Limitations

The readers are cautioned against over-interpreting this study results because this study has several limitations. First, the severity and patterns of psychosocial stresses were unclear; these stresses might relate to other causes of psychiatric disorders after newly diagnosed epilepsy. Second, this study excluded people younger than 20 years who were healthy, did not use health insurance and who were not covered by Taiwan’s National Health Insurance Program. Third, because the study did not have laboratory data, some potential clinical risk factors such as family history of epilepsy and psychiatric disorders could not be considered. Finally, because we used insurance claims from Taiwan’s National Health Insurance Research Database, ICD-9-CM coding defined mental disorders. This is a limitation because our results cannot be compared with previous and future studies which using ICD-10 or Diagnostic and Statistical Manual of Mental Disorders, Fourth Edition (DSM-IV) to define mental disorders. In order to improve upon the methodology used in this study, future studies may consider applying a population-based omnibus survey to better reflect epilepsy in the general population [53].

### Conclusion

We found an increased risk for a full spectrum of psychiatric disorders in newly diagnosed epilepsy patients, especially in the first year among the older patients and those with more previous consumption of epilepsy-related medical resources. These findings strongly suggest that newly diagnosed epileptic patients need access to mental health services, but further investigation will be needed to ascertain the causes of these phenomena.
